# Metastatic neuroendocrine pancreatic tumor – Case report

**Published:** 2018

**Authors:** EC Radu, AI Saizu, RR Grigorescu, AE Croitoru, C Gheorghe

**Affiliations:** * Gastroenterology Department, Fundeni Clinical Institute, Bucharest, Romania “Carol Davila” University of Medicine and Pharmacy, Bucharest, Romania

**Keywords:** pancreas, neuroendocrine, cancer

## Abstract

**Rationale.**Pancreatic neuroendocrine tumors (NETs) are rare neoplasms that develop from the endocrine tissues of the pancreas. They have a better overall prognosis than pancreatic adenocarcinoma. However, all commonly used classification systems reflect a separation between more indolent, well-differentiated tumors and far more aggressive poorly differentiated types that behave clinically more like small-cell carcinoma of the lung.

**Objective.**To present the case of a 62-year-old man with an aggressive pancreatic NET, with liver, splenic and bone metastases who underwent multidisciplinary treatment including several lines of chemotherapy, somatostatin analogs and radiotherapy.

**Methods and Results.**The patient is a smoker and an occasional drinker, known with type two diabetes mellitus (DM), receiving insulin therapy. He was diagnosed by contrast-enhanced computed tomography (CT) in January 2015 with a locally invasive pancreatic body mass, intraabdominal adenopathies and liver nodules, suggestive of metastases. Histopathological diagnosis was obtained through liver biopsy: neuroendocrine tumor with a 10-15% Ki67 proliferation index. Palliative chemotherapy with oxaliplatin and capecitabine was started in March 2015. In June 2015, Sandostatin LAR was added. In March 2016, he had progressive disease. Subsequently, in September 2016, bone metastasis was found within the T10 vertebra. He underwent radiotherapy for multiple bone metastases in February 2017. Progressive disease was again found during a CT examination in May 2017. His performance status has gradually worsened since then and he died in July 2017.

**Discussion.**As a group, well-differentiated gastroenteropancreatic NETs are generally indolent malignancies with prolonged natural history. Intermediate-grade NETs have a slightly worse prognosis than low-grade tumors.

**Abbreviations:** NETs – neuroendocrine tumors, NEC – neuroendocrine carcinoma, CT – computed tomography, MRI – magnetic resonance imaging, DM – diabetes mellitus, WHO – World Health Organisation, HCV – hepatitis C virus, CEA – carcinoembryonic antigen, AFP – alpha-fetoprotein, 5-HIAA – 5-Hydroxyindoleacetic acid, IHC – immunohistochemistry, EUS – endoscopic ultrasonography, EUS FNA – endoscopic ultrasonography with fine needle aspiration, CgA – chromogranin A, PRRT – peptide receptor radioligand therapy

## Introduction

More than 95 percent of the pancreatic neoplasms arise from the exocrine components. Neoplasms arising from the endocrine pancreas (neuroendocrine tumors, NETs) comprise less than 5 percent of pancreatic cancers. They can secrete a variety of peptide hormones, including insulin, gastrin, glucagon, and vasoactive intestinal peptide (VIP), resulting in multiple clinical syndromes. In modern clinical series, however, between 50 and 75 percent of pancreatic NETs are nonfunctioning. These tumors are characterized by variable but most often indolent biologic behavior. World Health Organization (WHO) classifies neuroendocrine neoplasms arising within the digestive system into two broad categories [**1**]:

• Well-differentiated NETs, further separated, according to the proliferative rate, into low-grade (G1) and intermediate grade (G2) subgroups. • Poorly differentiated neuroendocrine carcinomas (NECs) are all high-grade (G3) tumors. More recent publications show that morphological differentiation and Ki-67 are able to separate prognostic groups among G3 cases and therefore a separation of well-differentiated G3 NET with a Ki67 between 20-50%, from poorly differentiated G3 NEC with a Ki67 between 50-100% is emerging. This separation was shown to have clinical implications regarding the response to chemotherapy and survival [**2**][**3**].

The case of a well-differentiated metastatic pancreatic NET is described.

## Case presentation

The patient is a 62-year-old male, smoker (a 25 pack-year smoking history), an occasional alcohol drinker, without any family history of pancreatic or other types of malignancy. He is overweight (BMI=27.8), has high blood pressure and was diagnosed with type two diabetes mellitus (DM) in June 2014, receiving insulin therapy since then. He was first admitted to the Gastroenterology Department of Fundeni Clinical Institute on the 28th of January 2015. His complaints were: several episodes of acute diarrhea (3 stools/day) accompanied by a weight loss of 8 kg in the previous 3 months, diffuse, dull abdominal pain, aggravated by meals, occurring during day-time only, nausea and anorexia. Clinical examination revealed flushing and an enlarged, firm left liver lobe, with an irregular surface. Laboratory tests showed: hyperglycemia (15.71mmol/L), HbA1c=61mmol/mol, ALT=76U/L, total bilirubin=32.5µmol/L, ɣGT=634U/L, inflammatory syndrome with plasma fibrinogen=13.38µmol/L, negative HBs antigen and anti HCV (hepatitis C virus) antibodies, high CA19-9 (132.9UI/mL) and alpha-fetoprotein (30.7ng/mL), normal CEA (carcinoembryonic antigen).

**Fig. 1:**
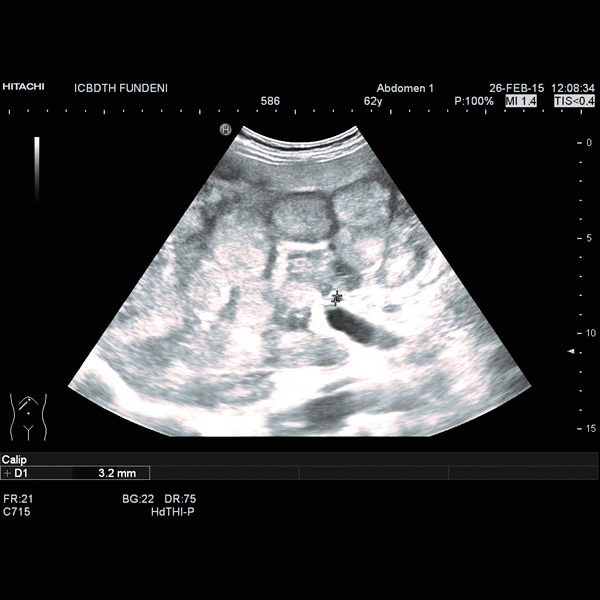
Multiple liver nodules, with hypoechoic rim (halo sign).

**Fig. 2:**
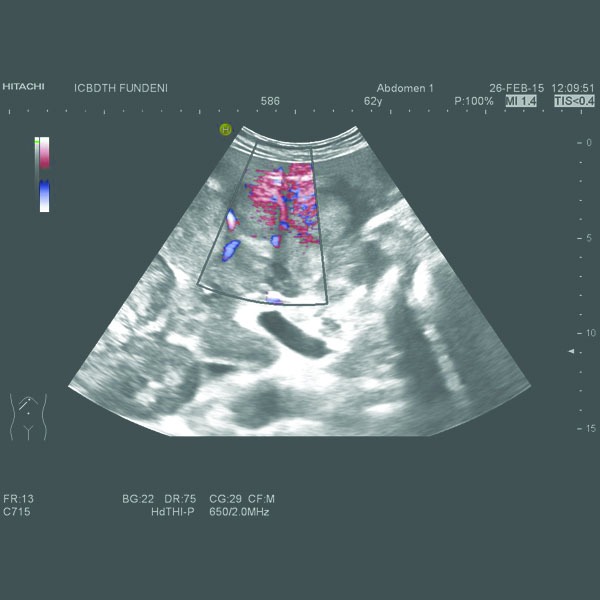
Hypervascular liver metastases.

A computed tomography (CT) had already been performed in another clinical unit and revealed a locally invasive tumoral mass at the level of the pancreatic body, 75/37 mm in size, without a clear demarcation line between the tumor, the posterior wall of the antrum and gastric body and the celiac trunk and its bifurcation. An enlarged liver with an irregular border and multiple nodules (maximum diameter of 63/44 mm) disseminated throughout the parenchyma, spontaneously hypodense, without intrahepatic bile duct dilation and retroperitoneal adenopathies (maximum size of 25 mm) were also described. The abdominal ultrasound we performed confirmed these findings (**Figures 1**, **2**).

Due to the high clinical suspicion of pancreatic NET, the level of chromogranin A (CgA) in the blood level was tested: 317μg/L (N: 19-98µg/L). The serotonin blood and urine level and the urinary 5-HIAA (5-Hydroxyindoleacetic acid) were normal. We also checked the blood levels of hormones like gastrin, glucagon and VIP, thinking they could be responsible for inducing diarrhea in this patient, in the case of a functioning pancreatic NET; they were all in the normal range.

**Fig. 3:**
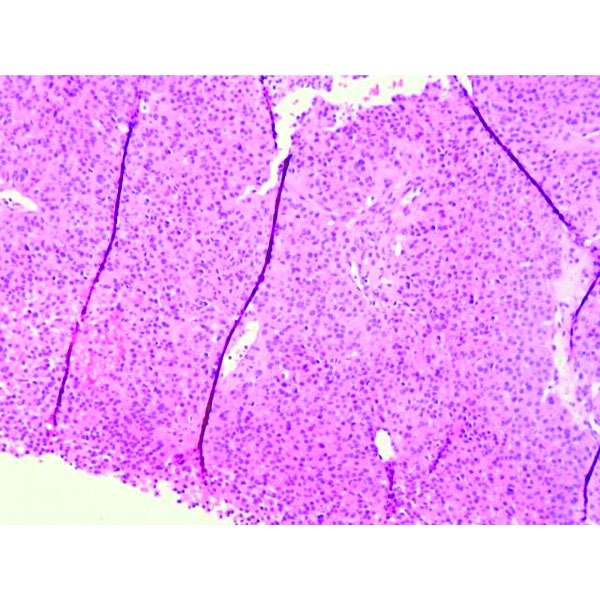
Hematoxylin and eosin staining. Atypical small cell proliferation.

**Fig. 4:**
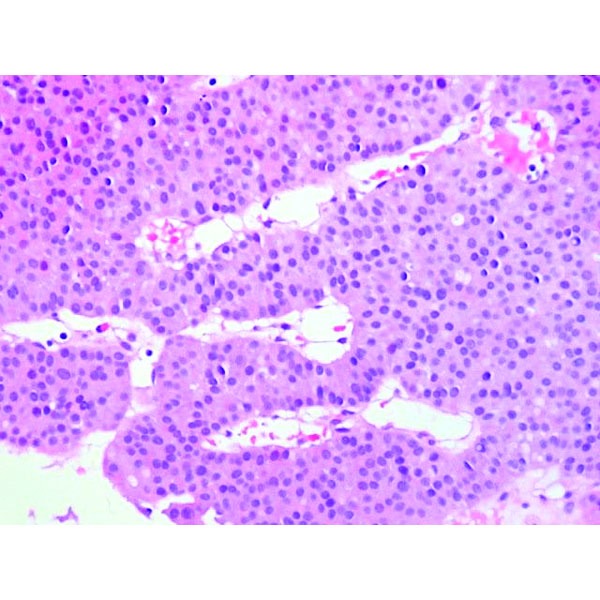
Monomorphic cells, with round nuclei and eosinophilic cytoplasm.

Percutaneous liver biopsy from one of the nodules was obtained. The histopathological examination described atypical small and monomorphic cell proliferation, with round nuclei, without obvious nucleolus, with eosinophilic cytoplasm; compact cellular placement, frequently around neoformed capillaries resulting in microacinar structures (**Figures 3**, **4**).

**Fig. 5:**
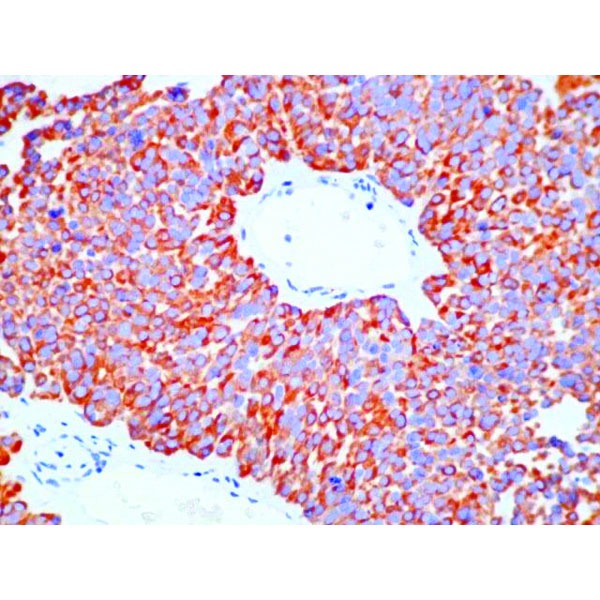
IHC positive for chromogranin A

**Fig. 6:**
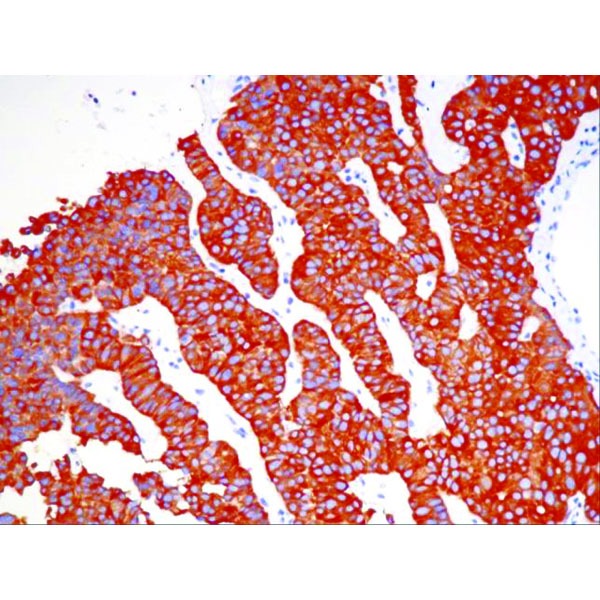
IHC positive for synaptophysin

**Fig. 7:**
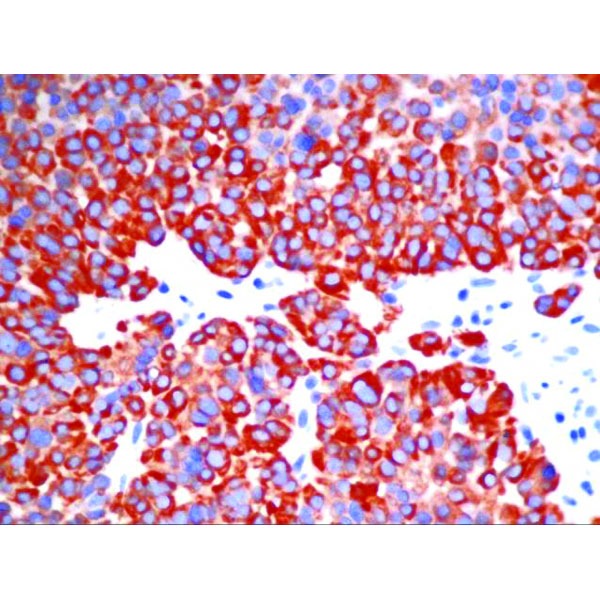
IHC positive for synaptophysin – detail

**Fig. 8:**
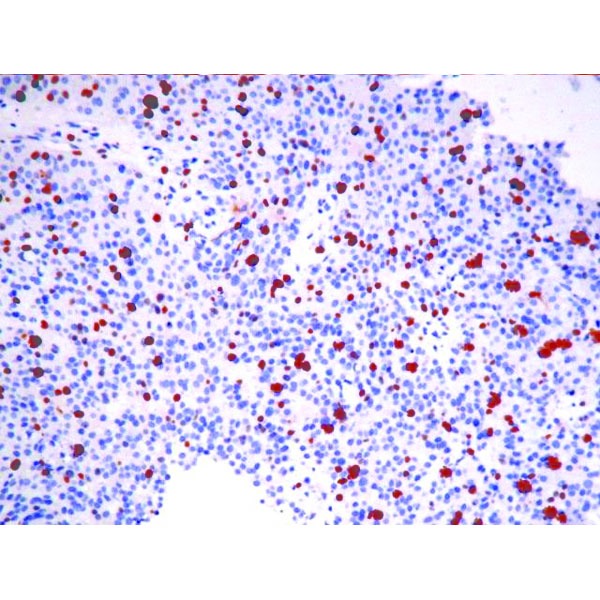
IHC for Ki67+ (10-15%)

Immunohistochemistry (IHC) was positive for CgA, strongly positive for synaptophysin, negative for CDX2 and TTF1, positive for AE1-AE3, with a Ki-67 proliferation marker of 10-15% in the hotspot areas (**Figures 5**, **6**, **7**, **8**). In conclusion, according to the “European Neuroendocrine Tumor Society/WHO nomenclature and classification for digestive system neuroendocrine tumors”, the diagnosis was intermediate grade (G2) well-differentiated pancreatic NET, with liver metastases.

The patient began palliative chemotherapy with capecitabine and oxaliplatin in March 2015. In June 2015, Sandostatin LAR 30 mg/week was added, and oxaliplatin was discontinued until March 2016 when the CT scan showed progressive disease. At that time oxaliplatin was reintroduced, and the dose of Sandostatin LAR was raised to 40mg/week, in an attempt to control tumor growth. In July 2016, he had stable disease following imaging assessment.

Because the patient complained of lower back pain, a bone scan was conducted in September 2016 and described hypermetabolic lesions of the T10 vertebra, probably malignant. At that time, zolendronic acid treatment was associated. An abdominopelvic MRI (magnetic resonance imaging) performed in November 2016 found multiple bone metastases at the level of the thoracolumbar vertebrae, with bilateral pre/paravertebral and also epidural extension. In December 2016,. The CT scan showed progressive disease again, with liver, splenic and bone metastases. Palliative radiotherapy was recommended and carried out in February 2017: a total dose of 20Gy in 4 fractions over 4 days at the T10-T11 level. In June 2017, he was admitted with abdominal pain, extreme fatigue, performance status ECOG=3 and cachexia. He received major pain control therapy and nutritional support. On June 29th, he received Nordic FOLFIRI chemotherapy. His condition progressively worsened, and he died on July 18, 2017.

## Discussion

Pancreatic NETs represent approximately 3 percent of primary pancreatic neoplasms. The nomenclature of pancreatic NETs has evolved considerably over the last twenty years. The term "pancreatic carcinoid" is no longer widely used. In recent years, the term "pancreatic neuroendocrine tumor" has been adopted by most practitioners as well as by the American Joint Committee on Cancer [**4**] and WHO [**5**]. The term “pancreatic neuroendocrine carcinoma” is reserved for those cases with poorly differentiated histology and a high proliferative rate.

Incidence rates have been increasing throughout the world over the last two decades, but it is likely that this is mainly related to increased detection of asymptomatic disease on cross-sectional imaging and endoscopy performed for other reasons [**6**][**7**]. Although they may manifest at any age, they most frequently occur in the fourth to sixth decades of life.

Potential risk factors for pancreatic NETs include diabetes (although the cause versus effect relationship is not clear), smoking (albeit with weak evidence), a previous history of chronic pancreatitis and genetic factors (NETs can be associated with hereditary endocrinopathies, including multiple endocrine neoplasia types I [**8**]. Our patient had several of these risk factors – smoking and a recent onset of DM.

Clinical presentation depends on whether the tumor is functional or not. Functioning tumors present with a specific hormonal syndrome (e.g., insulinoma, glucagonoma, gastrinoma, VIPoma). Although nonfunctioning pancreatic NETs do secrete some substances such as chromogranins, neuron-specific enolase, pancreatic polypeptide, and ghrelin, they do not present clinically with a hormonal syndrome. As a result, they are often diagnosed later in the course of the disease with symptoms of local compression or metastatic disease. In a variety of reports, between 32 and 73 percent of cases are metastatic at the moment of diagnosis [**7**][**9**]. The most common site of metastatic disease involvement for pancreatic NET is the liver [**10**].

Chromogranin A is the most commonly secreted and measured hormone associated with all types of gastroenteropancreatic NETs. CgA can be followed as a tumor marker if elevated at baseline, as was the case in our patient. False positive elevations of CgA can be seen in patients taking proton pump inhibitors. For patients with functioning pancreatic NETs, the levels of the secreted hormone represent a more specific tumor marker. In our patient, serotonin, 5-HIAA, glucagon, VIP and gastrin levels were normal.

Modern imaging modalities have led to significant improvement in the localization and accurate staging of pancreatic NETs. High-resolution cross-sectional CT or MRI scanning is highly sensitive for the identification of primary pancreatic NETs as well as liver metastases. Early arterial phase imaging is particularly valuable for the detection of hypervascular tumors [**11**].

Diagnostic imaging using radiolabeled somatostatin analogs offers whole-body imaging and functional information regarding tumoral expression of somatostatin receptors and is potentially useful for targeted therapy. Somatostatin receptor imaging was not performed in our case because the access to octreoscan in our country is very limited; there are only two locations where it can be performed and it is not covered by the public health insurance system. The decision to start Sandostatin treatment was based on multiple reasons: well-differentiated pancreatic NET, with high tumor burden, studies showing that the majority of pancreatic NETs present somatostatin receptors subtype 2 with anti-proliferative effect.

Endoscopic ultrasonography (EUS) is highly sensitive to occult, subcentimeter pancreatic NETs detection, and it plays an important role in the evaluation of patients with functional NETs undetectable using conventional imaging techniques [**12**]. Endoscopic ultrasound with fine needle aspiration (EUS FNA) also offers the ability to obtain a histopathologic diagnosis. In our case, the diagnosis was reached by percutaneous liver biopsy, the risk of bleeding during EUS FNA being considered too high (the primary tumor was in the vicinity of large vessels).

Intermediate-grade gastroenteropancreatic NETs, like in our patient, have a slightly worse prognosis than low-grade tumors do [**13**]. Although they are classified and treated similarly at present, as new treatment modalities become available, it is likely that the histological grade (G) of a well-differentiated NET will affect the selection of the appropriate treatment.

For those who have a potentially resectable metastatic disease, resection may provide prolonged control of symptoms and tumor growth. However, the majority of patients present with tumor recurrence. For patients with unresectable disease, options to control tumor growth and symptoms related to tumor bulk or hormonal hypersecretion include somatostatin analogs, nonsurgical liver-directed therapy (e.g., hepatic artery embolization), and systemic antitumor therapy. Our patient received a somatostatin analog in combination with systemic therapy. Patients with symptoms of hormone hypersecretion should be managed with somatostatin analogs and other agents corresponding to the specific syndrome. Studies have also demonstrated an antiproliferative effect of somatostatin analogs in metastatic NETs [**14**]. For metastatic pancreatic NETs, reports suggest an antitumor activity for some oxaliplatin-based regimens: the outcomes in 16 patients treated with capecitabine, oxaliplatin and bevacizumab were 18.8 percent with a partial response and 69 percent with stable disease [**15**]. Other options include temozolomide or streptozocin-based regimens and molecularly targeted agents, like everolimus and sunitinib. The choice of initial chemotherapy, in this case, was based on the available options in our clinic, at that time. We considered oxaliplatin therapy to be the most suitable, even if it is usually used for G3 tumors, due to the aggressive clinical pattern of the disease (liver and lymph node metastases) and to the lack of better options. Everolimus is not available in our country. Sunitinib and capecitaine/temozolomide were accessible only in clinical trials, for a limited period.

The role of peptide receptor radioligand therapy (PRRT) in patients with progressive advanced pancreatic NET remains investigational. However, when available, targeted radiotherapy using radiolabeled somatostatin analogs is a reasonable approach for patients with somatostatin receptor-positive disease, otherwise refractory to medical therapy. PRRT is not available in Romania.

## Conclusion

Pancreatic NETs are a heterogeneous group and treatment should be established by a multidisciplinary team, considering factors related to the tumor stage and behavior.

**Sources of Funding**

None

**Disclosures**

The authors declare that there is no conflict of interest.
